# Epigenetic Regulation of NK Cell-Mediated Antitumor Immunity

**DOI:** 10.3389/fimmu.2021.672328

**Published:** 2021-05-04

**Authors:** Miaoran Xia, Bingbing Wang, Zihan Wang, Xulong Zhang, Xi Wang

**Affiliations:** ^1^ Department of Immunology, School of Basic Medical Sciences, Capital Medical University, Beijing, China; ^2^ Advanced Innovation Center for Human Brain Protection, Capital Medical University, Beijing, China; ^3^ Beijing Key Laboratory for Cancer Invasion and Metastasis Research, Capital Medical University, Beijing, China; ^4^ Department of Oncology, Capital Medical University, Beijing, China

**Keywords:** natural killer (NK) cells, epigenetics, DNA methylation, histone modification, transcription factor, microRNA, antitumor immunity

## Abstract

Natural killer (NK) cells are critical innate lymphocytes that can directly kill target cells without prior immunization. NK cell activation is controlled by the balance of multiple germline-encoded activating and inhibitory receptors. NK cells are a heterogeneous and plastic population displaying a broad spectrum of functional states (resting, activating, memory, repressed, and exhausted). In this review, we present an overview of the epigenetic regulation of NK cell-mediated antitumor immunity, including DNA methylation, histone modification, transcription factor changes, and microRNA expression. NK cell-based immunotherapy has been recognized as a promising strategy to treat cancer. Since epigenetic alterations are reversible and druggable, these studies will help identify new ways to enhance NK cell-mediated antitumor cytotoxicity by targeting intrinsic epigenetic regulators alone or in combination with other strategies.

## Introduction

Natural killer (NK) cells are potent effector lymphocytes of the innate immune system. They serve as the first line of defense against infected or transformed cells without prior sensitization. Compared with T and B cells, which recognize targets by their antigen-specific cell surface receptors (TCRs/BCRs), NK cell activation is controlled by the balance between activating and inhibitory signals from multiple germline-encoded receptors. These cells patrol for potential target cells that lack major histocompatibility complex class I (MHC I) or overexpress ligands to activate NK cell receptors (NCRs) (1). NK cells are initially recruited to the tumor microenvironment (TME) during the tumor killing process and then are activated by complex signals arising from multiple ligand-receptor interactions. Activated NK cells release cytotoxic granules containing perforin and granzyme B upon forming an immunological synapse with the target cells (2). Perforin forms pores in the membrane of target cells, thus allowing granzymes to enter the cell and initiate cell death (3, 4). NK cells can also induce cell apoptosis through the engagement of Fas ligands (FasL) or tumor necrosis factor-related apoptosis-inducing ligands (TRAIL) with Fas and TRAIL receptors on tumor cells (5, 6). In a process known as antibody-dependent cell cytotoxicity, NK cells recognize opsonized tumor cells *via* Fc receptors (CD16) and kill them by releasing cytolytic granules. Lysis leads to an increased release of tumor antigens and further primes adaptive immune responses. In addition to direct cytotoxic activity, NK cells can function as central communicators of innate and adaptive immunity in the TME by secreting multiple chemokines (CCL3, CCL4, CCL5, and XCL1), cytokines (IFN-γ, TGF-β, and IL-10), and growth factors (GM-CSF) (7). In this way, these cells communicate with various immune cells within tumor tissues, including monocytes, granulocytes, dendritic cells, T cells, and stromal cells (8).

NK cells play important roles in cancer immunosurveillance, particularly by eliminating early tumors and metastasis (minimal disease). In 1970s, several groups found non-MHC-restricted antitumor activity of NK cells in mice (9–12). Later, the rapid and potent cytotoxicity of NK cells against target cells was also observed in humans (13). Furthermore, an eleven-year follow-up study found that the impaired NK cell killing capacity in the peripheral blood is correlated with tumor incidence and prognosis (14). Compared with the role of T cells in antitumor immunity and adoptive cellular therapy, NK cells have certain advantages and greater potential “off-the-shelf” utility (7). They are as effective as T cells (15, 16) but less toxic because they cause fewer immune-related adverse events. Mature NK cells are effector cells with a broader reactivity to tumors due to their independent recognition of specific receptors and antigen presentation by MHC molecules. Their lytic responses can be triggered within minutes without clone selection and differentiation (1). The “ready-to-go” state is associated with the unique epigenetic features of NK cells, as shown in the following sections.

## NK Cell Plasticity

NK cells are a heterogeneous and plastic population. They are classically defined as CD3^-^CD56^+^ cells in humans and divided into two major subsets, CD56^dim^CD16^+^ and CD56^bright^CD16^low^ (17–19). CD56^dim^CD16^+^ subsets are highly cytotoxic effector cells that are predominantly found in peripheral blood. CD56^bright^CD16^low^ subsets are recognized as immature NK cells with immune regulation functions through cytokine secretion. They preferentially reside in secondary lymphoid organs, such as lymph nodes. The surface markers of murine NK cells vary depending on the mouse strain. In C57B/6 and SJL mice, NK cells express NK1.1, NKp46, and CD49b (2). For other strains, such as BALB/c, NK cells express CD49b and NKp46 while possessing allelic variants of NK1.1 (2). Tumor necrosis factor receptor superfamily member CD27 and the integrin CD11b are used to mark NK cell differentiation in mice. The most cytotoxic NK cells are recognized as CD27^-^CD11b^+^, regulatory NK cells are CD27^+^CD11b^+^, and immature NK cells are CD27^+^CD11b^-^ (20, 21).

NK cells belong to the family of innate lymphoid cells (ILCs). NK cells and ILC1s are grouped into group I innate lymphoid cells (22). ILC1s reside in tissues and function as cytokine secretors. Conventional NK (cNK) cells and ILCs arise from distinct progenitors (23). However, many surface markers initially described on NK cells, such as CD122, NK1.1, and NKp46, can be expressed on ILC1s (24). The mixed phenotype can be explained by imprinting the effects of the tissue microenvironment and cell activation state. Therefore, at present, the definition of NK cells based on their phenotype is essentially at a steady state (24). The majority of human mature NK cells can be identified as CD3^-^CD127^-^CD7^+^CD56^+^ (or NKp46^+^)T-bet^+^Eomes^+^ lymphocytes, and mature mouse NK cells can be identified as CD3^−^CD127^−^NK1.1^+^ (or NKp46^+^)T-bet^+^Eomes^+^ lymphocytes. There are no markers that can unambiguously distinguish NK cells and ILC1s in human or mouse tissues during infection or inflammation (25).

The conversion between NK cells and ILC1s in the TME was recently described (26). Transforming growth factor-β (TGF-β) in the TME could drive NK cells (CD49a^−^CD49b^+^Eomes^+^) to convert into intermediate ILC1 (intILC1, CD49a^+^CD49b^+^Eomes^+^) populations and ILC1 (CD49a^+^CD49b^−^Eomes^int^) populations. IntILC1s and ILC1s are less cytotoxic and cannot control local tumor growth and metastasis (27). SMAD4, which is a unique common SMAD, acts as a central mediator that facilitates the canonical TGF-β signaling pathway (28). TGF-β induces salivary gland ILC differentiation by suppressing Eomes through a JNK-dependent, Smad4-independent pathway (29). However, *Smad4* deficiency does not affect ILC1 differentiation but surprisingly alters the phenotype of cNK cells. Cortez et al. reported that *Smad4*-deficient NK cells showed features of ILC1s and lost effector functions to control tumor metastasis. Mechanistically, SMAD4 restrained noncanonical TGF-β signaling mediated by the cytokine receptor TGFβR1 in NK cells (30). A subsequent study by Wang et al. showed that selective deletion of *Smad4* in NK cells led to impaired NK cell maturation, NK cell homeostasis, and NK cell immune surveillance against melanoma metastases and cytomegalovirus. These changes were associated with a downregulation of granzyme B (*Gzmb*), *Kit*, and *Prdm1* in *Smad4*-deficient NK cells and independent of canonical TCF-β signaling (31).

Of note, it has become increasingly clear that various subsets of tissue-resident NK (trNK) cells exist, which differ from cNK cells in their origin, development, and function (32–34). Unlike circulating and widely distributed cNK cells, trNK cells were found to populate multiple tissue sites, including the liver, lung, skin, uterus, salivary gland, adipose tissue, and kidneys (32). trNK cells are distinct from cNK cells in the expression of surface markers and transcription factors. For example, murine liver trNK (LrNK) cells express relatively low levels of NK cell maturation-associated markers, such as CD11b, CD49b (DX5), and Ly49 receptors (35). The development of LrNK is independent of Eomes, while T-bet, Hobit, PLZF, and AhR are more critical for LrNK cell development than cNK cells (34). trNK cells are actively involved in multiple processes, such as antiviral infection, mediating immune tolerance, and promoting fetal growth (34). The accumulation of LrNK cells in hepatocellular carcinoma patients is correlated with poor prognosis (36), suggesting a potential role in tumor development. More comprehensive studies are needed to investigate the role of trNK in antitumor immunity.

Although historically known as innate lymphoid cells, NK cells can also achieve memory characteristics similar to those of adaptive immune cells, such as antigen specificity, longevity, and enhanced recall responses. Memory NK responses were first reported in mouse models of anti-murine cytomegalovirus (MCMV) infection (37) and delayed hypersensitivity reactions to chemical haptens and viral antigens (38, 39). During secondary MCMV infection, memory NK cells bearing the virus-specific Ly49H receptor can rapidly proliferate, degranulate and produce cytokines by recognizing the MCMV-encoded glycoprotein m157 (37). Memory NK cells have also been described in humans expressing NKG2C in HCMV-seropositive individuals (40). Growing evidence suggests that memory-like NK cell responses may occur in response to a broader range of viral, bacterial, and even eukaryotic pathogens (41). The responses of memory-like NK cells against tumors are poorly understood, and two key questions remain to be answered: (1) whether NK cells can acquire memory properties during the antitumor process and (2) whether memory NK cells from infection models can acquire stronger *in vivo* killing capacity targeting tumor cells.

Compared with cNK cells that live less than ten days (42, 43), memory NK cells can persist for years in some individuals and are important for controlling CMV throughout life (44, 45). Similar to CD8^+^ T cells, NK cells also exhibit an “exhausted” phenotype in individuals with malignancies or chronic viral infections. This phenotype is represented by a loss of activating receptors (e.g., NKG2D) and increased expression of checkpoint receptors (e.g., NKG2A, TIGIT, PD-1, TIM-3, LAG-3), which severely impair their antitumor function (46). Compared with the “suppression” state, which is reversible after the withdrawal of inhibitory signaling, the “exhaustion” state is not transient and undergoes stable epigenetic changes (47). Antagonistic antibodies (Abs) (e.g., anti-PD-1, anti-TIGIT, and anti-NKG2A monoclonal Abs) can recover NK cell antitumor capacity (46, 48). However, epigenetic intervention should be considered to reactivate exhausted NK cells intrinsically in future studies.

## Epigenetic Regulators Modulating NK Cell-Based Antitumor Immunity

Epigenetic alterations are reversible and heritable changes that do not alter DNA sequences, including DNA methylation, posttranslational modifications of histone proteins, changes in transcription factors, and noncoding RNA expression. Despite the deep understanding of NK cell biology, research on epigenetic regulation of NK cell function is just beginning. In this review, we provide an overview of the epigenetic regulators that modulate NK cell-based antitumor immunity, and the findings will hopefully help to identify novel approaches and potential targets for tumor immunotherapy.

### DNA Methylation

**Figure 1:**
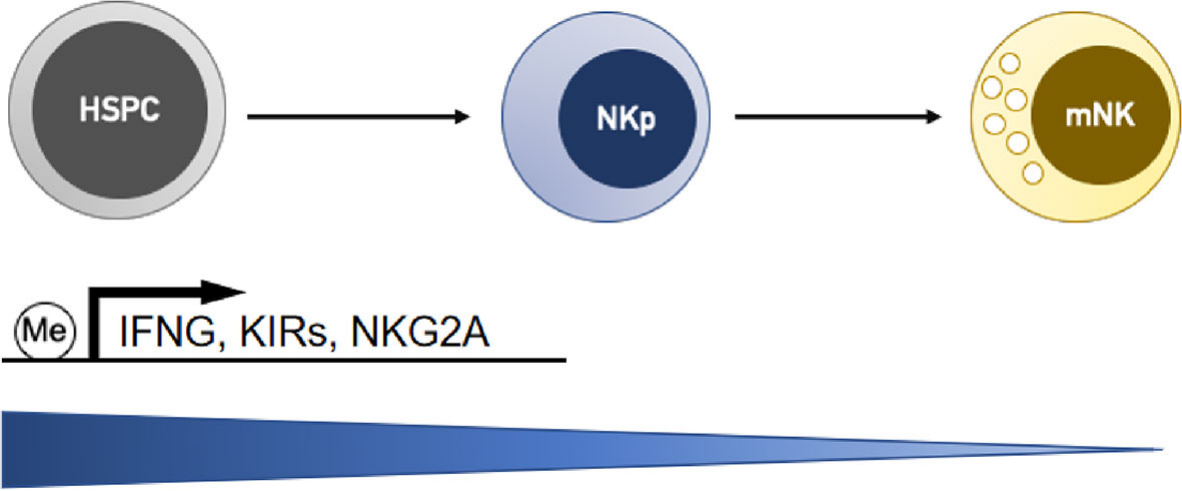
NK cells gradually downregulate DNA methylation levels at the gene promoters of interferon-γ (IFNG) and receptors (KIRs and NKG2A) during the differentiation process, and this activity is correlated with the upregulation of their transcription. HSPC, hemopoietic stem/progenitor cells; NKp, NK cell progenitors; mNK, mature NK cells.

DNA methylation is a heritable epigenetic marker that correlates with gene repression. During the terminal differentiation process, NK cells gradually acquire the ability to produce IFN-γ through demethylation and epigenetic remodeling at the IFNG promoter (**Figure 1**) (49). DNA methylation has been reported to correlate with the gene expression of a variety of NK cell receptors, including killer Ig-like receptors (KIRs) and natural cytotoxic receptors (NCRs). KIRs are polymorphic groups of molecules, and some are expressed while others are silenced in the same cell. Different KIRs can transmit inhibitory or activating signals to NK cells, and effector function is considered to result from the balance of these contributing signals. The expression repertoire of KIRs is critical for NK killing ability. Moderate demethylation of the inhibitory KIR promoter is essential for normal NK recognition and lysis of abnormal cells. Promoter methylation of KIR genes consistently silences KIR expression (50, 51) and chromatin is condensed in early hemopoietic progenitor cells. During NK cell differentiation and maturation, the chromatin structure opens, and KIR genes sequentially become demethylated and transcribed (**Figure 1**) (52). Excessive demethylation of the inhibitory KIR promoter represses NK cytolytic function and results in tumor escape. Some studies demonstrated that acute exercise could cause promoter demethylation of the activating NK-cell receptor KIR2DS4 (53) and changed DNA methylation in 33 targets (25 genes) (54). Of the targets, 19 showed decreased methylation and 14 showed increased methylation. Whether these changes lead to functional adaptations needs to be elucidated. In addition, DNA methylation is crucial in maintaining the allele-specific expression of the inhibitory receptor NKG2A. CpGs are methylated in NKG2A-negative stages (hemopoietic stem cells, NK progenitors, and NKG2A-negative NK cells) but hypomethylated specifically in various developmental stages of NKG2A-positive NK cells and NK cell lines (**Figure 1**) (55). Natural killer group 2 member D (NKG2D) is one of the most crucial activating receptors of NK cells for target recognition. The methylation frequency of the NKG2D promoter can be used as a biomarker for detecting hepatitis B virus-associated hepatocellular carcinoma (HCC). NKG2D promoter methylation in HCC patients was higher than that in chronic hepatitis B patients and healthy controls (56).

Hypomethylating agents 5-azacytidine (5-aza) and decitabine (Deci) are approved for the treatment of acute myeloid leukemia (AML) and myelodysplastic syndrome (MDS). However, the direct effect of demethylating treatment on NK cell function remains controversial (**Table 1**) and should be considered in the application of these drugs. Both 5-aza and Deci can alter the expression of KIRs on NK cells and may thus affect NK reactivity against malignant hematopoietic cells (57–59). Demethylation treatment with 5-aza significantly suppresses the cytolytic activity of the NK-92MI cell line and human polyclonal NK cells, which is related to the overexpression of inhibitory KIRs and impaired granzyme B (GzmB) and perforin (Prf1) release by these cells (57, 58). However, another study reported that systemic treatment with 5-aza leads to an increased proportion of Ki-67^+^ NK cells expressing multiple KIRs in MDS patients. These proliferating NK cells exhibit increased IFN-γ production and degranulation towards tumor target cells (59). However, Kubler et al. found that low-dose and long-term treatment of humanized NSG mice with 5-aza does not induce common inhibitory KIR expression but instead promotes the differentiation of various NK-cell precursor subsets to enhance the antitumor (pediatric BCP-ALL *in vivo*) response (60). The different effects could be determined based on the dose, with high doses of the demethylating agents showing cytotoxicity and lower doses mediating DNA hypomethylation. Deci decreases NK cell cytotoxicity at intermediate concentrations and leads to a U-shaped dose-response curve (0-20 μM). In contrast, increased inhibitory KIRs (KIR3DL1, KIR2DL1, KIR2DL2/DL3), decreased NKG2D, and increased NKp44 expression have been induced by Deci treatment in a linear dose-response manner (61). However, another group reported that low-dose Deci (0.2 mg/kg) reduces the antitumor response of NK cells in tumor-bearing mice (75), and Deci has also been shown to increase the cell surface expression of recombinant UL16 binding protein (ULBP) (62) and MHC class I-related molecule B (MICB) (63), the ligands of NKG2D in AML cells, and the NKG2D-dependent sensitivity of these cells to NK-mediated killing *in vitro*.

**Table 1:** Epigenetic drugs targeting DNA methylation and histone modification related to NK antitumor cytotoxicity.

Agents		Effects	NK cytotoxicity	References
Hypomethylating agent	5-aza	↑inhibitory KIRs	↓	(57, 58)
		↓granzyme B and perforin release		
		↑Ki-67^+^ NK cells	↑	(59)
		↑IFN-γ production		
		↑degranulation		
		- inhibitory KIRs	↑	(60)
		↑NK precursor differentiation		
	Deci	↑inhibitory KIRs	U-shaped response (lowest at intermediate dose)	(61)
		↓NKG2D expression		
		↑NKp44 expression		
		↑NKG2DL (ULBP and MICB) on AML cells	↑	(62, 63)
HATi	Curcumin	↓NKG2D transcription	↓	(64)
		↓NKG2D-dependent NK cell degranulation and IFN-γ secretion		
HDACi	Entinostat (class I HDACi)	↑MIC expression, Death receptors and PD-L1 expression on tumor targets	↑	(65, 66)
		↑NKG2D expression		
	SAHA (Pan-HDACi)	- degranulation	↓	(67)
	Panobinostat	↓NKG2D, CD16 and NKp46 expression	↓	(67)
		↓degranulation		
	Romidepsin	- NKG2D, CD16 and NKp46 expression	↓	(67)
		↓degranulation		
	TSA	↓NK degranulation	↓	(68, 69)
	(Pan-HDACi)	↓IFN-γ production		
	VPA	↓NKG2D and NKp46 expression on resting NK cells		
	(class I and IIa HDACi)	↓NKG2D, NKp44 and NKp46 expression on NK cells stimulated with IL-12, IL-15 and IL-18		
	NaB			
	(class I and IIa HDACi)			
Histone methylase inhibitor	UNC1999	↑NK degranulation	↑	(70)
	EPZ005687	↑CD122 & NKG2D on NK cells		
	(EZH2 inhibitor)			
	GSK343	↑NKG2D-Ligand on tumor cell surface	↑	(71)
	GSK126			
	(EZH2 inhibitor)			
	GSK-J4	↓IFN-γ,TNFα,GM-CSF and IL-10	–	(72)
	(JMJD3/UTX inhibitor )	↓granzyme B, perforin, NCRs, ULBPs in mRNA level		
Histone demethylase inhibitor	SP-2509	↓NK cell metabolism	↓	(73, 74)
	SP-2577			
	(scaffolding LSD1 inhibitor)			

↑, up-regulated; ↓, down-regulated; -, unchanged.

5-aza, 5-azacytidine; KIRs, killer immunoglobulin-like receptors; IFN-γ ; interferon-γ; Deci, decitabine; NKG2DL, NKG2D ligands; ULBP, UL16-binding protein; MICAB, MHC class I chain-related gene B; AML, acute myeloid leukemia; HATi, histone acetyltransferases inhibitor; HDACi, histone deacetylases inhibitor; PD-L1, programmed death ligand-1; SAHA, suberoylanilide hydroxamic acid; TSA, trichostatin A; VPA, valproic acid; NaB, sodium butyrate; EZH2, enhancer of zeste homolog 2; NCR; natural cytotoxicity receptors; JMJD3, jumonji domain-containing protein D3; TNFα, tumor necrosis factor-alpha; GM-CSF, granulocyte-macrophage colony-stimulating factor; LSD1, lysine-specific histone demethylase 1.

### Histone Modification

Histone modifications are associated with the opening or closing state of the chromatin structure, which results in the activation or repression of gene transcription (76). Of particular importance are histone acetylation and methylation. The acetylation of lysine residues on histone 3 (AcH3) and 4 (AcH4) is associated with active transcription (77), while methylation contributes to both active and suppressed states of gene expression. The methylation of histone 3 lysine 9 (H3K9) and H3K27 is inhibitory, whereas the methylation of H3K4, H3K36, and H3K79 is activating (78). The level of histone modification is controlled by the interplay between enzymes: e.g., histone acetyltransferases (HATs) vs. deacetylases (HDACs) (79) and histone methyltransferases vs. demethylases. The dynamic histone modification states determine NK cell activation and effector function in antitumor immunity (80).

#### Histone Acetylation

Histone acetylation precedes the transcription of many genes (e.g., IFNG and NKG2D) involved in regulating NK cell function (81–83). Chang et al. compared long-range histone hyperacetylation patterns across the *Ifng* gene region in T cells and NK cells and found that histone acetylation of the *Ifng* gene depends on stimulation and the transcription factors Stat4 and T-bet in T cells. In contrast, even in resting NK cells, histones along *Ifng* gene region are already acetylated, and additional proximal domains are hyperacetylated after stimulation of transcription (84). These characteristics may partially explain the quick response of NK cells without prior sensitization. The NKL cell line exhibits high levels of AcH3, AcH4, and H3K4me3 in the NKG2D gene. A significantly high level of AcH3, especially H3K9ac, was observed in the NKG2D gene of NK cells from peripheral blood, while a low level of H3K4me3 was present. Repressive histone modifications (H3K27me3 and H3K9me2) to the NKG2D gene in both NKL and peripheral NK cells were hardly detectable (64).

HAT inhibitor (curcumin) incubation reduced H3K9Ac levels of the NKG2D gene, downregulated NKG2D transcription, and led to a marked reduction in NKG2D-dependent NK cell degranulation and IFN-γ secretion by NKL cells (64). HDAC inhibitors (HDACis) have emerged as novel immunomodulatory drugs and have been reported to affect NK cell cytotoxicity against tumors through both receptor and ligand modulation. The expression of activating ligands for NK cell recognition was increased after HDACi treatment on the cell surfaces of neuroblastoma, melanoma, osteosarcoma, colon, and Merkel cell carcinomas (65, 85). However, different HDAC inhibitors were reported to have varying effects on the NK cell phenotype (**Table 1**). There are four subclasses of HDACs (HDAC I, II, III, IV). Treatment with a histone deacetylase inhibitor (trichostatin A, TSA) alone was sufficient to induce inhibitory NKG2A receptor expression in mice (55). Entinostat (a class I HDACi) treatment induced NK activation *via* increased MIC expression in tumor targets as well as enhanced NKG2D expression and ADCC-mediated lysis in primary human NK cells (65, 66). Many HDACis have been reported to negatively regulate the NK antitumor response, including vorinostat (SAHA), panobinostat, romidepsin, TSA, valproic acid (VPA), and sodium butyrate (NaB) (**Table 1**) (67). They affect NK cell activation through cytokine receptors and activating receptors involved in tumor cell recognition (68, 69). The inhibitory effect on nuclear mobilization of p50 and NK-κB activation caused by HDAC inhibitors also resulted in impaired NK cell activation (82).

#### Histone Methylation

Li et al. screened 4 upregulated (KMT2C, KDM6B, UTY, and JARID2) and 4 downregulated (ASH1L, PRMT2, KDM2B, and KDM4B) histone methyltransferases/demethylases upon activation of human NK cells by gene expression profiling, which was further confirmed by qPCR and western blot in NK92MI cells. These enzymes were mainly associated with H3K4 methylation and H3K27 methylation, and they only affected limited gene loci instead of the global modification state. Bivalent marks with both H3K4me3 and H3K27me3 determined the “poised” chromatin state of many genes associated with NK activation. This state helps the rapid shift in expression above the baseline during the target recognition process. Treatment with UNC1999 could induce NK cell degranulation. In addition, the expression of IFN-γ and TNF-α is increased after treatment with OG-L002 and MM102 (80).

Histone lysine N-methyltransferase Ezh2 (enhancer of zeste homolog 2) contributes to histone repressive marks H3K27me3. Loss of Ezh2 or inhibition of its enzymatic activity with small molecules in both mouse and human hematopoietic stem and progenitor cells enhanced NK cell expansion and cytotoxicity against tumor cells through upregulation of CD122 and NKG2D (**Table 1**) (70). The Ezh2 inhibitor EPZ011989 and combination treatment with cisplatin in HT1376 (bladder cancer cell line) xenografts led to increased expression of CD86, MIP-1α, and CD3d at the transcript level as well as CD56 and NCR1 at the protein level, indicating an active state of NK cells (86). Ezh2 was also found to be a transcriptional repressor of NKG2D ligands. Ezh2 inhibition enhanced NK cell eradication of tumor cells in hepatocellular carcinoma (**Table 1**) (71). Jumonji-type histone H3K27 demethylases (e.g., JMJD3/UTX) have been identified as key regulators of cytokine production in human NK cell subsets. The JMJD3/UTX inhibitor GSK-J4 increased global levels of the repressive H3K27me3 mark around the transcription starting site (TSS) of effector cytokine genes. However, NK cell cytotoxic killing activity against tumor cells was unaffected after treatment with GSK-J4 (**Table 1**) (72).

Methylation of H3K4 is an activating mark for gene transcription. An H3K4me1-marked latent enhancer at the *Ifng* locus was essential for NK memory in a systemic endotoxemia model (87). The H3K4me3 demethylase Kdm5a associates with p50 and binds to the suppressor of cytokine signaling 1 (Socs1) promoter region in resting NK cells, thus leading to a repressive chromatin configuration. Kdm5a deficiency impairs the activation of NK cells, leading to decreased IFN-γ production and impaired phosphorylation and nuclear localization of STAT4 (88). LSD1 is a histone demethylase of H3K4me1/2 and H3K9me1/2. Catalytic LSD1 inhibitors blocking demethylase activity are unaffected on NK cells, while scaffolding inhibitors disrupting epigenetic complexes, including LSD1, impair NK cell metabolism and cytotoxicity through depletion of glutathione (**Table 1**) (73, 74).

### Transcription Factors

**Figure 2:**
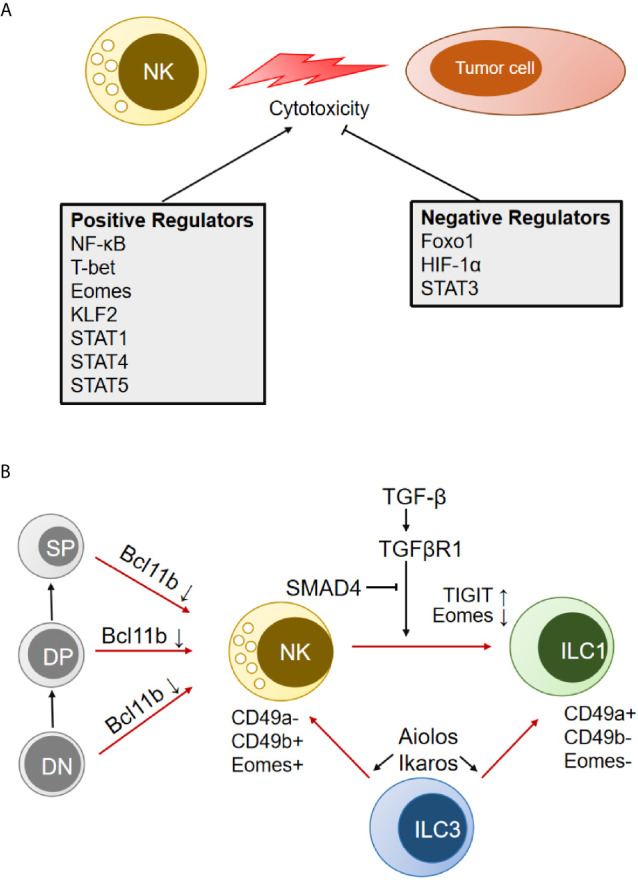
Transcription factors (TFs) that modulate NK cell cytotoxicity and transdifferentiation. **(A)** TFs that positively and negatively regulate NK antitumor cytotoxicity are indicated separately. **(B)** Schematic representation of multiple TFs involved in the transdifferentiation between NK cells and other immune cells. DN, double-negative cells in the thymus; DP, double-positive cells in the thymus; SP, single-positive cells in the thymus; ILC, innate lymphoid cells.

Transcription factors (TFs) are specific kinds of proteins that can activate or suppress the transcriptional activity of target DNA sequences by specifically recognizing and binding them. Many TFs have been shown to highly modulate the function of human or murine NK cells and affect the eradication of tumor cells (**Figure 2A**) (89–91). Kwon HJ et al. reported that silencing the expression of the NF-κB p65 subunit caused a significant reduction in the mRNA levels of IFN-γ, TNF-α, MIP-1α/β, GramB, and IκBα induced by NKG2D and 2B4 coengagement (92). The T-box transcription factors T-bet and Eomes are both critical in driving the differentiation and function of NK cells (93). T-bet deficiency impairs the longevity and function of NK cells in inhibiting cancer metastasis, which further precludes the initiation of a potent adaptive response to tumors in mice. Adoptive transfer of wild-type activated NK cells (but not T-bet^-/-^ NK cells) protects T-bet^-/-^ animals after melanoma challenge (94). Aiolos is required for the maturation of CD11b^+^CD27^-^ NK cells. However, NK cells lacking Aiolos are strongly hyperreactive to various NK cell-mediated tumor models but impaired in controlling viral infection (95). Foxo1 was identified as a negative intrinsic regulator of NK cell homing, late-stage maturation, and effector functions, and it can directly target IFN-γ expression; moreover, Foxo1 deficiency increases the NK cell killing capacity of tumor cells *ex vivo* and the antimetastatic activity *in vivo*. Foxo1 suppresses Tbx21 expression through direct binding to its promoter in human NK cells and through association with the promoter *via* recruitment by Sp1 in murine NK cells (96). Phosphorylation-mediated inactivation of Foxo1 facilitates the activating receptor CD226 regulation of NK cell antitumor responses (97). Krupple-like factor 2 (KLF2) is a key TF responsible for expanding transferred NK cells and prolonging their functionality within the tumor. KLF2 imprints a homeostatic pattern on mature NK cells that allows them to migrate to IL-15-rich microenvironments (98). Cells adapt to hypoxia in solid tumors by upregulating HIF-1α. Inhibition of HIF-1α unleashes the antitumor activity of human tumor-infiltrating NK cells associated with high expression of IFN-γ in an IL-18-dependent manner (99).

It has been reported that the signal transducer and activator of transcription (STAT) family (STAT1, STAT3, STAT4, STAT5) positively or negatively regulates NK cell activity (**Figure 2A**) (100). STAT1 dysfunction in humans and genetic deletion in mice leads to impaired NK cell antitumor cytotoxicity (101). Mutation of the S727 phosphorylation site of STAT1 (Stat1-S727A) increases the expression of perforin and granzyme B and enhances NK cell cytotoxicity in various tumor models, including for melanoma, leukemia, and metastasizing breast cancer. Inhibition of upstream cyclin-dependent kinase 8 (CDK8) may be a therapeutic strategy for stimulating NK cell-mediated tumor surveillance (102). Full-length STAT1α is efficient for NK cell maturation and tumor control in mice, while NK cells from the C-terminally truncated STAT1β isoform show impaired maturation and effector functions (103). STAT-3 regulates all aspects of NK biology, including almost all of the pathways for target cell killing and the reciprocal regulatory interaction between NK cells and other components of the immune system, which has been presented in detail by Nicholas A. Cacalono (104). STAT4 signaling in NK cells could be activated by IL-2 (105) and IL-12 (106), which specifically bind to the human perforin gene and induce activation of NK antitumor activity. Eckelhart et al. found that STAT5^fl/fl^ Ncr1-iCreTag mice show a marked reduction in NK cells in the spleen and lymph nodes and severely impaired NK-dependent antitumor activity (107). There are two homologs of STAT5, STAT5A and STAT5B, which can form homos, heterodimers, and tetramers. It was reported that the loss of STAT5B (but not STAT5A) reduces NK cell numbers and cytotoxicity (108). However, recent studies have shown that STAT5A deficiency is sufficient to compromise NK cell homeostasis, responsiveness, and tumoricidal function (109, 110).

In addition, several TFs have been shown to control the transdifferentiation between NK cells and other immune cells (T cells, ILCs) (**Figure 2B**). Downregulation of Eomes by TGF-β signaling in the TME could induce the conversion of mouse NK cells to an NK-ILC1 intermediate cell type (intILC1s) and, finally, to ILC1s, which are less cytotoxic and cannot control local tumor growth and metastasis (27). Cortez et al. found that SMAD4 is a negative regulator of NK-ILC1s conversion in a noncanonical TGF-β signaling pathway (30). SMAD4 is the only common SMAD in TGF-β signaling that usually impedes immune cell activation in the tumor microenvironment. Selective deletion of Smad4 in NK cells impairs tumor cell rejection, promotes tumor cell metastases, and impedes NK cell homeostasis and maturation. GzmB was identified as a direct target of a transcriptional complex formed by SMAD4 and JUNB (31). It was also found that ILC3 could transdifferentiate into IFN-γ-producing ILC1 and NK cells by IL-1β plus IL-12 stimulation, which is associated with the upregulation of T-bet and Aiolos. Degradation of Aiolos and Ikaros proteins by lenalidomide inhibits ILC1/NK cell transdifferentiation and ILC1/NK cell function (111). Bcl11b, a zinc finger transcription factor, is essential for the maintenance of T-cell identity. Upon Bcl11b deletion, immature thymic T cells could convert to NK cells and acquire NK cell properties (112, 113). The converted NK cells were called T-to-natural killer (ITNK) cells and exhibited enhanced antitumor activity. They are considered an attractive cell source for cancer immunotherapy (114).

### miRNA

**Figure 3:**
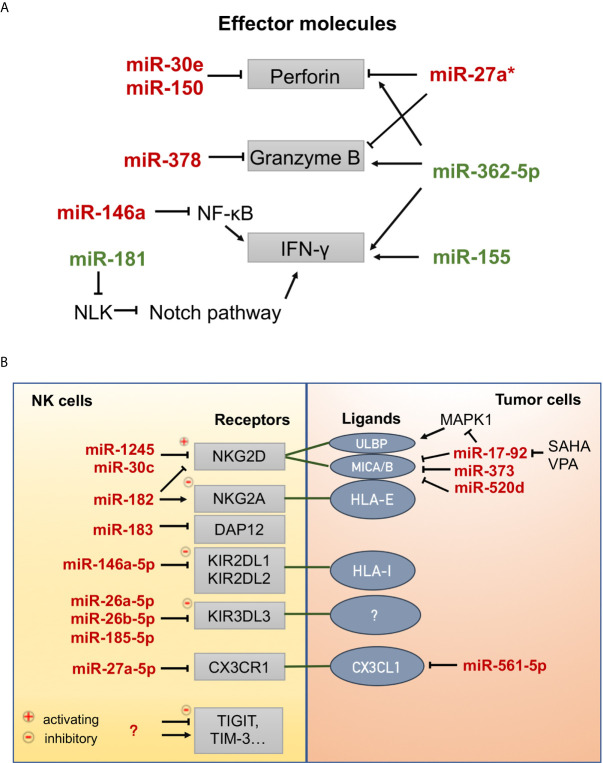
MicroRNAs involved in the effector functions of NK cells. **(A)** MicroRNAs that positively (green) or negatively (red) regulate the expression of effector molecules (perforin, granzyme B, and interferon-γ). NLK: nemo-like kinase, Notch signaling inhibitor. **(B)** MicroRNAs that regulate the expression of receptors on NK cells and ligands on tumor cells. CX3CR1, C-X3-C motif chemokine receptor 1; CX3CL1, C-X3-C motif chemokine ligand 1; DAP12, DNAX-activating protein 12 kDa, an exclusive signaling adaptor of many NK cell receptors; HLA-I, human leukocyte antigen, class I; HLA-E, human leukocyte antigen, Class I, E; KIR2DL1, killer cell immunoglobulin-like receptor, two Ig domains and long cytoplasmic tail 1; KIR2DL2, killer cell immunoglobulin-like receptor, two Ig domains and long cytoplasmic tail 2; KIR3DL3, killer cell immunoglobulin-like receptor, three Ig domains and long cytoplasmic tail 3; MICA/B, MHC class I-related molecule A/B; NKG2A, natural-killer group 2 member A; NKG2D, natural-killer group 2 member D; SAHA, suberoylanilide hydroxamic acid (vorinostat), histone deacetylase inhibitor; TIGIT, T cell immunoreceptor with Ig and ITIM domains; TIM-3, T cell immunoglobulin and mucin domain-containing protein 3; ULBP, UL16 binding protein; VPA, valproic acid, histone deacetylase inhibitor.

MicroRNAs (miRNAs) are small single-stranded noncoding RNAs that target mRNA and promote degradation by binding to the 3’ untranslated region (UTR) (115). miRNAs can modulate gene expression involved in the development, maturation, and effector functions of NK cells (**Figure 3**) (116).

Prf1 and GzmB are the main effector molecules of NK cells. Prf1 could be targeted by miR-30e (117) and miR-150 (118), GzmB could be targeted by miR-378 (117), while both could be targeted directly by miR-27a* (119) in resting and activated states and indirectly by miR-27a-5p (120) by downregulating the expression of C-X3-C motif chemokine receptor 1 (CX3CR1) under TGF-β1 signaling. Tumor cells upregulate miR-561-5p, which in turn inhibits the production of CX3CL1 and subsequently reduces NK cell recruitment to the tumor (**Figure 3A**) (121). Wang et al. reported that miR-146a negatively regulates IFN-γ production in human NK cells by targeting the NK-κB signaling pathway (**Figure 3A**) (122). MiR-146a overexpression significantly suppresses the cytotoxic activity of NK92 cells by targeting STAT1 signal transduction (123). In contrast, miR-181 was found to promote IFN-γ production in primary NK cells in response to cytokine stimulation by targeting nemo-like kinase (NLK), an inhibitor of Notch signaling (124). MiR-362-5p overexpression upregulated Prf1, GzmB, IFN-γ, and CD107a in human NK cells (125). Several reports have shown that miR-155 can enhance NK cell functions by regulating molecules involved in NK cell activation and IFN-γ release (126–128).

Moreover, miRNAs can control the expression of activating and inhibitory receptors on the surface of NK cells or that of their ligands on tumor cells (**Figure 3B**). Human miR-1245 could downregulate NKG2D on NK cells and, therefore, impair NKG2D-mediated functions of NK cells (129). NKG2D ligands (MICA/B) could also be repressed by miR-20a, miR-93, miR-106b, miR-373, and miR-520d in human cancer cells (HeLa, 293T, DU145, and glioma cells) (130, 131). In breast cancer cells, the miR-17-92 cluster (miR-20a, miR-20b, miR-93, and miR-106b), which could be inhibited by the HDAC inhibitors SAHA and VPA, downregulates the expression of MICA/B by targeting the mRNA 3’-UTR and downregulates ULBP2 by inhibiting the MAPK/ERK signaling pathway (132). The transcription and translation of DNAX-activating protein 12 kDa (DAP12), an exclusive signaling adaptor of many NK cell receptors, could be repressed by human miR-183, thus leading to the abrogation of NK cell antitumor function (133). In contrast, miR-30c-1* (134) promotes NK cell cytotoxicity against hepatoma cells by targeting the transcription factor HMBOX1 and miR-30c (135) could promote the cytotoxicity of NKL cells *in vitro* by upregulating the expression levels of NKG2D, CD107a, and FasL. Inhibitory receptors (e.g., KIRs, NKG2A, PD-1, TIGIT, TIM-3) function as immune checkpoints associated with NK cell exhaustion and the immune escape of tumor cells. MiR-146a-5p can downregulate the expression of both KIR2DL1 and KIR2DL2 (136). Three miRNAs, miR-26a-5p, miR-26b-5p, and miR-185-5p, were identified as inhibitors of the expression of inhibitory KIR3DL3, whose function has not yet been demonstrated (137). MiR-182 mediates a complex modulation of NKG2D and NKG2A levels at different stages of human hepatocellular carcinoma, resulting in increased Prf1 expression (138). Some miRNAs have been found to target PD-1 [miR-28 (139), miR-138 (140), miR-4717 (141)] and TIM-3 [miR-28 (139)] in T cells and cause T cell exhaustion. Thus, these miRNAs may also play a regulatory role in NK cells; however, experimental evidence has not been presented.

## Perspectives

NK cells play a crucial role in preventing tumor initiation and metastasis. Many studies have illustrated the epigenetic regulatory mechanism of NK cell antitumor cytotoxicity, and they mainly focused on the expression of NK cell receptors and effector molecules, as we reviewed above. Multiple modulators always participate in epigenetic regulation. For example, histone modifications determine the open/closed state of chromatin, which affects the binding of transcription factors to specific regulatory sites. Additional research should focus on the interactions between different epigenetic modulators rather than just studying individual molecules. Recent technological advances have allowed us to gain a deeper understanding of NK cells. For example, single-cell RNA sequencing helps decipher the similarities and differences between humans and mice and between blood and splenic NK cells (142). Very recently, Li et al. applied the transposase accessible chromatin with sequencing (ATAC-seq) technique to define two distinct TF clusters that dynamically regulate NK cell differentiation in a homemade *in vitro* NK cell differentiation system (143). NK cells are a heterogeneous population that consists of multiple subsets and various states. The tissue site shapes the functional potential of NK cell subsets. Whole transcriptome profiling reveals the site-specific variations of NK cells in the lymph node, lung, blood, bone marrow, and spleen (33). However, the epigenetic features of these subsets are still a mystery.

The “states” (resting, activating, memory, repressed, and exhausted) of NK cells are controlled epigenetically, although insights into the underlying mechanism are very limited. Adaptive NK cells exhibit a unique whole-genome epigenetic signature similar to that of effector memory CD8^+^ T cells but not conventional NK cells (144). Chronic stimulation (NKG2C Abs with IL-15) could induce exhaustion in primary adaptive NK cells, thereby upregulating the expression of checkpoint receptors LAG-3 and PD-1. These NK cells are dysfunctional when challenged with tumor targets and exhibit a whole genome-DNA methylation profile similar to the epigenetically remodeled profiles of exhausted CD8^+^ T cells (145). It is reasonable to presume that NK cells are similar to T cells and show susceptibility to exhaustion during the antitumor war. However, there is a lack of consensus on the defining features of NK cell dysfunctional states, such as senescence, suppression, and exhaustion (47). Further consideration is needed to determine the state of NK cells in the antitumor response and how their epigenetic landscape changes during the process.

NK cell-based immunotherapy is an effective supplement to T cell-based therapy. Various approaches have been introduced to activate NK cells in adoptive cell therapy for better clinical outcomes, including generating CAR-NKs and inducing ADCC by mAbs, immune checkpoint blockade, engineered cytokine stimulatory, and so on (146). Even so, NK cell-based therapies are still in the early stages of development. Other than these “extrinsic” strategies, approaches that target “intrinsic” epigenetic regulators should be taken into consideration. Research on the epigenetic control of NK cell functions will provide new evidence for developing drugs and effective cancer prevention approaches. For example, demethylating agents can restore the absence of transcription of NKG2DL associated with high levels of DNA methylation in tumor cells. Some histone modification regulators (e.g., EZH2 and LSD1) have been found to be aberrantly overexpressed in various malignant tumors. Small molecular inhibitors are in clinical or preclinical development. From our perspective, these inhibitors also have potential applications in improving the *in vitro* expansion of NK cell cytotoxicity. More studies are needed to further elucidate the application of epigenetic drugs in NK cell-based immunotherapy, alone or in combination with other strategies.

## Author Contributions

MX and XW conceived and designed the manuscript. MX did literature searching, drafted the manuscript, and drew the figures. BW and ZW did literature searching and drafted several sections. XZ and XW reviewed and revised the article. All authors contributed to the article and approved the submitted version.

## Funding

This work was supported by grants from the Scientific Research Common Program of Beijing Municipal Commission of Education (KM201910025026 to MX), the Support Project of High-level Teachers in Beijing Municipal Universities in the Period of 13th Five–year Plan (IDHT20190510 to XW and XZ), and the National Natural Science Foundation of China (81972652 to XW).

## Conflict of Interest

The authors declare that the research was conducted in the absence of any commercial or financial relationships that could be construed as a potential conflict of interest.
